# A comprehensive study to the assessment of Arrhenius activation energy and binary chemical reaction in swirling flow

**DOI:** 10.1038/s41598-020-64712-y

**Published:** 2020-05-12

**Authors:** Noor Saeed Khan, Zahir Shah, Meshal Shutaywi, Poom Kumam, Phatiphat Thounthong

**Affiliations:** 1 Department of Mathematics, Abdul Wali Khan University, Mardan, 23200 Khyber Pakhtunkhwa Pakistan; 2 Department of Mathematics College of Science & Arts, Rabigh King Abdul-Aziz University, Rabigh, 21911 Saudi Arabia; 3 KMUTT Fixed Point Research Laboratory, Room SCL 802 Fixed Point Laboratory, Science Laboratory Building, Department of Mathematics, Faculty of Science, King Mongkut’s University of Technology Thonburi (KMUTT), Bangkok, 10140 Thailand; 4 KMUTT-Fixed Point Theory and Applications Research Group, Theoretical and Computational Science Center (TaCS), Science Laboratory Building, Faculty of Science, King Mongkut’s University of Technology Thonburi (KMUTT), Bangkok, 10140 Thailand; 5 Department of Medical Research, China Medical University Hospital, China Medical University, Taichung 40402 Taiwan; 6 Renewable Energy Research Centre, Department of Teacher Training in Electrical Engineering, Faculty of Technical Education, King Mongkut’s University of Technology North Bangkok, 1518, Wongsawang, Bangsue, Bangkok, 10800 Thailand

**Keywords:** Physics, Mathematics and computing

## Abstract

Nanotechnology research has a huge impact upon biomedicine and at the forefront of this area are micro and nano devices that use active/controlled motion. In this connection, it is focus to investigate steady three dimensional rotating flow with heat and mass transfer incorporating gyrotactic microorganisms. Buongiorno’s nanofluid formulation is followed for thermophoresis and Brownian motion, porous space, Arrhenius activation energy and binary chemical reaction with some other effects. An enhanced analytical method is applied to solve the nondimensional equations. The non-dimensional parameters effects on the fields of velocity, temperature, nanoparticles concentration and gyrotactic microorganisms concentration are shown graphically. Velocity decreases while temperature and nanoparticles concentration increase with magnetic field strength. Gyrotatic microorganisms motion becomes slow with rotation parameter. Due to rotation, the present problem can be applied in microbial fuel cells, food processing, microbiology, biotechnology and environmental sciences, electric power generating and turbine systems, computer disk drives, mass spectromentries and jet motors.

## Introduction

Energy conservation is the voice of the day. All the old methods which restored the energy resources or storage are given up due to the speed of modern life requirements. It is required that to have more energy on account of less expenditures of raw materials which are producer of less byproduct in the form of environmental pollution. In these days scientists and researchers consider nanotechnology as the best option to have all the potentials of present time energy conservations. Nanotechnology rests on nanoparticles made of metallic, non-metallic, carbide or oxide materials having the radius in 100 nm. Choi^1^ was the first one who opened the door of nanotechnology by working on nanofluid. Nanofluids have the tonic role when used with microorganisms to provide useful products for life and to eradicate the serious environmental issues. Al-Khaled *et al*.^2^ studied theoretically the application of bioconvection phenomena in periodically flow of tangent hyperbolic nanofluid over an accelerated moving surface with nonlinear thermal radiation, chemical reaction, thermophoresis and Brownian motion. Khan *et al*.^3^ used convective Nield boundary conditions to investigate the rheology of couple stress nanofluid with activation energy, porous media, thermal radiation, gyrotactic microorganisms employing Buongiorno nanofluid model, in addition to, second-order velocity slip (Wu’s slip). Tlili *et al*.^4^ presented a novel study about the flow, heat and mass transfer as well as motile microorganisms of magnetohydrodynamic Oldroyd-B nanofluid past a stretching cylinder. Alwatban *et al*.^5^ explained the rheological aspects of Eyring Powell nanofluid past a moving surface where velocity decreases with magnetic force and porous medium while non-Newtonian parameter has opposite effects on velocity. Waqas *et al*.^6^ worked on numerical side of stretching flow of micropolar nanofluid with microorganisms, activation energy and convective Nield boundary conditions implementing shooting method. Waqas *et al*.^7^ also organized a project to deliver the explorations on Maxwell viscoelasticity-based micropolar nanofluid with porous media using MATLAB bvp4c package where velocity increases with slip and micro-rotation parameters. Khan *et al*.^8^ reflected on most gains achieved by including copper nanomaterial in the base fluid. Highest volumes were witnessed in conductivity. Zuhra *et al*.^9^ estimated the revenue on graphene nanoparticles used for the thermal conductivity. Cloud enhancement rose with the addition of nanopartices. Nanofluid and thermodynamic literature can also exists in the literature with refs. ^10–19^.

Rotating flows have applications in formulating the conditions inside the wheel spacing of gas turbines as well as in rotating cavity to model the conditions between compressor disks or co-rotating turbines, thin film fluid flow through a rotating surface, conical diffuser circulative flow, impinging jet disk cooling, shrouded rotation of disks, contra-rotating disks for wheel space in contra-rotating disks of existing engines, gears, bearings, rolling elements, polymer processing, lubrication systems etc. Khan *et al*.^20^ provided a sharp entrant into the rapid rotating business which has played catch up with profiles such as flow, heat transfer, chemical reactions and entropy generation. Ahmad *et al*.^21^ paid attention to the nanofluid whose thermal conductivity jumped on higher quantity as the nanoparticles rise, while a short-covering rally in rotating flow is also added. Hayat *et al*.^22^ treated Arrhenius activation energy and binary chemical reaction, irreversibility, heat generation/absorption, viscous dissipation, Brownian motion, thermophoresis in the thermodynamics of Ree-Eyring fluid with nanomaterials in two rotating disks. Li *et al*.^23^ at bioconvection rotating flow opened on a positive note and started to write that exact solutions are obtained analytically for the nonlinear phenomena and the study could provide a theoretical base for comprehending the transportation of unsteady bioconvection. Hayat *et al*.^24^ among the key sectors, presented exploration that has rotating linked benefits while flow rate are also remained higher on higher quantity of relevant parameter.

Fluid flows in porous media have numerous applications in environmental sciences and industries like ground water systems, erection of oil reservoirs in insulating systems, geothermal energy systems, heat exchange layouts, nuclear waste disposal, catalytic reactors, flow of water in reservoirs *etc*. Khan *et al*.^25^ shared the index gained for flow and heat transfer at high values of parameters where thermal system shows that as many as parameters were active all of them declined the profile. Rahman *et al*.^26^ disclosed that the heating volumes stood high as compared with the turn over of magnetic field parameter quantities. Heat quantifies sharply higher led by suction parameter depreciation in the thermal system while pressure remained also higher for nanoparticles. Khan *et al*.^27^ analyzed the Darcy law for porous medium to show the effects on flow and heat transfer of second-grade fluid. Zuhra *et al*.^28^ worked on porous medium to investigate the flow of gyrotatic microorganisms and homogeneous-heterogeneous chemical reactions with buoyancy effects. Khan *et al*.^29^ reported the role of porous medium in second-grade liquid film flow and heat transfer with entropy generation, chemical reaction and stratification. Palwasha *et al*.^30^ discussed porous medium for simultaneous flow and heat transfer in two non-Newtonian nanoliquids with gyrotactic microorganisms and nanoparticles. Khan *et al*.^31^ presented the porous medium behavior for MHD second-grade nanofluid flow, heat and mass transfer as well as gyrotactic microorganisms in gravity driven problem.

Microorganisms have played a vital role in improving the human beings life, especially, due to the applications on medical side. Without the useful microorganisms, life is impossible to lead. These organisms are too small to see even through a powerful microscope but do big for the environment. Their participation in life is in biofuels, industrial and environmental systems, enzyme biosensors, mass transportations, biotechnology and biological sciences. Researchers have deep interest to work on microorganisms. Khan *et al*.^32^ reported a likely surge in nanoparticles and motile organisms transports supporting parametric study. Positive impact of gyrotactic microorganisms fall on the systems denominated by fluid flow. Zuhra *et al*.^33^ presented a study that stands for the thermal system decline due to higher assigned values of energy parameter of slip. Khan *et al*.^34^ assembled conclusions on liquid velocity and heating transportation with small organisms as sharp valuation in systems takes place on account of gyrotactic microorganisms. Zuhra *et al*.^35^ expected more gains achieved through following the gyrotactic microorganisms for convective instability enhancement possibly facilitating the conduction. Khan *et al*.^36^ presented the bioconvection in nanofluid flow in rotating system with entropy generation which shows that gyrotatic microorganisms flow is reduced with increasing the rotation parameter.

Arrhenius activation energy (AAE) is the minimum energy required to start the chemical reaction on which pioneered work is of Arrhenius in 1889. On acquiring the AAE, the particles (atoms, molecules) are ready to take part in chemical reaction. AAE has applications in oil and pharmaceutical industries, MHD, environmental and geothermal systems. More studies and applications of AAE and binary chemical reactions (BCR) are already discussed in the studies with refs. ^3–6,11,22^.

To discuss AAE and BCR with bioconvection due to gyrotactic microorganisms in rotating systems of two disks is still require explorations. So, the present study reflects highest gains on including, movements, heating capability, saturation and gyrotactic microorganisms due to Arrhenius activation energy and binary chemical reaction via optimal homotopy analysis method^22,37^.

## Method

### Formulation

**Figure 1:**
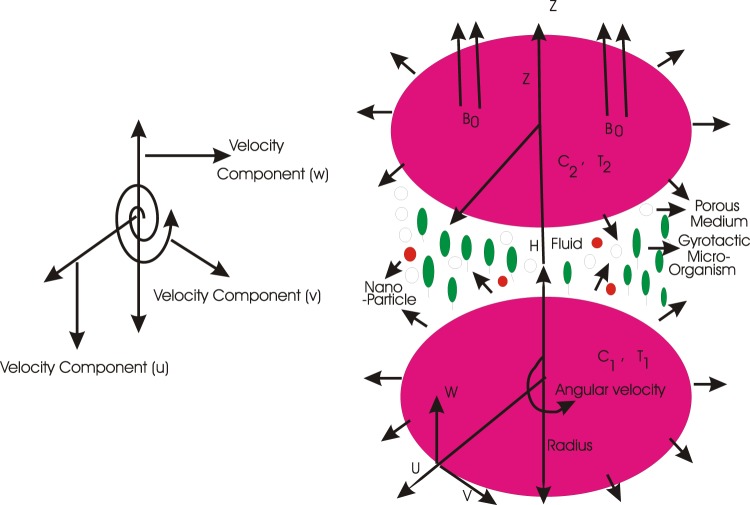
Geometry of the problem.

A revolving movement of magnetized, time non-reliant and lack of compressible nanodispersion in three dimensions is under focused in the persistence of porous region, AAE and BCR. A below disc is situated at *z* equal to zero. Both the discs are at a distance *H* apart. The speed of below and upper discs are respectively Ω_1_ and Ω_2_. Similarly their expanding values are respectively *a*_1_ and *a*_2_. Magnetic environment also exists carrying the power *B*_0_ along with the *z*-side (please consult to Fig. 1).

For the life of microorganisms, aquas exits as the background dispersion accompanying nanoparticles. The temperatures, tiny particles concentrations and gyrotactic microorganisms are (*T*_1_, *T*_2_), (*C*_1_, *C*_2_) and (*N*_1_, *N*_2_) on the respective disks. The tiny particles saturation on both the disks are obeyed by the actively confined formulation *i. e*. there exist the tiny particles motion at the walls. Consideration is taken for the tiny particles dispersion that the background dispersion is strong which keeps nothing with the swimming direction as well as movement of the small organisms. The below several profile statements carrying the preservation of grand amount of matter, movement, heating notion, tiny particles saturation, accompanying small organisms are given as in^23^

1 $$\nabla \cdot {\bf{v}}=0,$$

2 $${\rho }_{f}({\bf{v}}\cdot \nabla )\nabla \cdot {\bf{v}}=-\nabla p+{\mu }_{f}{\nabla }^{2}{\bf{v}},$$

3 $${(\rho c)}_{P}({\bf{v}}\cdot \nabla )T=\alpha {\nabla }^{2}T+\tau \left[\begin{array}{c}{D}_{B}\nabla T\cdot \nabla C+\left(\begin{array}{c}\frac{{D}_{T}}{{T}_{2}}\end{array}\right)\nabla T\cdot \nabla T\end{array}\right],$$

4 $$({\bf{v}}\cdot \nabla )C={D}_{B}{\nabla }^{2}C+\left(\begin{array}{c}\frac{{D}_{T}}{{T}_{2}}\end{array}\right){\nabla }^{2}T,$$

5 $$\nabla \cdot {\bf{j}}=0,$$

where **v** = (*u*, *v*, *w*) manifests the velocity of the nanodispersion, *C* manifests the tiny particle saturation, *ρ*_*f*_ manifests the tiny dispersion density, *P* manifests force per unit area, *μ*_*f*_ manifests dynamic viscosity reliant to nanodispersion and small organisms, *α* manifests heating diffusion of the nanodispersion, $\tau =\frac{{(\rho c)}_{P}}{{(\rho c)}_{f}}$ in which (*ρc*)_*P*_ denotes the heating storage space of tiny particles and (*ρc*)_*f*_ denotes the heating storage space reliant to dispersion. The subscript “*f*” is used for the base fluid. *D*_*B*_ manifests the nanoparticles random motion diffusivity notation, *D*_*T*_ manifests the heat reliant diffusivity constant, **j** manifests the microorganisms flux defined as^23^

6 $${\bf{j}}=N{\bf{v}}+N\tilde{v}-{D}_{n}\nabla N,$$

notice that *N* manifests the distribution of small organisms, *D*_*n*_ manifests the diffusion of small organisms, $\tilde{v}$ manifests the mean rate of velocity of gyrotactic microorganisms which physical quantity having direction is defined as^23^

7 $$\tilde{v}=\left(\begin{array}{c}\frac{b{W}_{c}}{\Delta C}\end{array}\right)\nabla C,$$

notice that *b* manifests the chemotaxis nonvariable and *W*_*c*_ manifests the highest cell traveling motion.

Working on Eqs. (1–5), the velocity, heating, saturation and distribution of small organisms accompanying the effects of magnet environment, porous media, heat source/sink and activation energy with binary chemical reaction are as of a form of^20–25^

8 $$\frac{\partial u}{\partial r}+\frac{u}{r}+\frac{\partial w}{\partial z}=0,$$

9 $${\rho }_{f}\left(\begin{array}{c}u\frac{\partial u}{\partial r}+w\frac{\partial u}{\partial z}-\frac{{v}^{2}}{r}\end{array}\right)=-\frac{\partial p}{\partial r}+{\mu }_{f}\left(\begin{array}{c}\frac{1}{r}\frac{\partial u}{\partial r}-\frac{u}{{r}^{2}}+\frac{{\partial }^{2}u}{\partial {r}^{2}}+\frac{{\partial }^{2}u}{\partial {z}^{2}}\end{array}\right)-{\sigma }_{f}{B}_{0}^{2}u-\frac{{\mu }_{f}}{{k}_{0}}u,$$

10 $${\rho }_{f}\left(\begin{array}{c}u\frac{\partial v}{\partial r}+w\frac{\partial v}{\partial z}+\frac{uv}{r}\end{array}\right)={\mu }_{f}\left(\begin{array}{c}\frac{1}{r}\frac{\partial v}{\partial r}-\frac{v}{{r}^{2}}+\frac{{\partial }^{2}v}{\partial {r}^{2}}+\frac{{\partial }^{2}v}{\partial {z}^{2}}\end{array}\right)-{\sigma }_{f}{B}_{0}^{2}v-\frac{{\mu }_{f}}{{k}_{0}}v,$$

11 $${\rho }_{f}\left(\begin{array}{c}{\rm{u}}\frac{\partial w}{\partial r}+{\rm{w}}\frac{\partial w}{\partial z}\end{array}\right)=-\frac{\partial p}{\partial z}+{\mu }_{f}\left(\begin{array}{c}\frac{1}{r}\frac{\partial w}{\partial r}+\frac{{\partial }^{2}w}{\partial {r}^{2}}+\frac{{\partial }^{2}w}{\partial {z}^{2}}\end{array}\right)-\frac{{\mu }_{f}}{{k}_{0}}w,$$

12 $$\begin{array}{c}\left(\begin{array}{c}u\frac{\partial T}{\partial r}+w\frac{\partial T}{\partial z}\end{array}\right)=\alpha \left(\begin{array}{c}\frac{1}{r}\frac{\partial T}{\partial r}+\frac{{\partial }^{2}T}{\partial {r}^{2}}+\frac{{\partial }^{2}T}{\partial {z}^{2}}\end{array}\right)\\ \,+\tau \left[\begin{array}{c}{D}_{B}\left(\begin{array}{c}\frac{\partial T}{\partial r}\frac{\partial C}{\partial r}+\frac{\partial T}{\partial z}\frac{\partial C}{\partial z}\end{array}\right)+\frac{{D}_{T}}{{T}_{2}}{\left(\begin{array}{c}\frac{\partial T}{\partial r}\end{array}\right)}^{2}+{\left(\begin{array}{c}\frac{\partial T}{\partial z}\end{array}\right)}^{2}\end{array}\right]\\ \,+{\sigma }_{f}{B}_{0}^{2}({u}^{2}+{v}^{2})+{Q}_{0}(T-{T}_{2}),\end{array}$$

13 $$\begin{array}{c}u\frac{\partial C}{\partial r}+w\frac{\partial C}{\partial z}={D}_{B}\left(\begin{array}{c}\frac{1}{r}\frac{\partial C}{\partial r}+\frac{{\partial }^{2}C}{\partial {r}^{2}}+\frac{{\partial }^{2}C}{\partial {z}^{2}}\end{array}\right)+\frac{{D}_{T}}{{T}_{2}}\left(\begin{array}{c}\frac{1}{r}\frac{\partial T}{\partial r}+\frac{{\partial }^{2}T}{\partial {r}^{2}}+\frac{{\partial }^{2}T}{\partial {z}^{2}}\end{array}\right)\\ \,-{k}_{r}^{2}(C-{C}_{2}){\left[\begin{array}{c}\frac{T}{{T}_{\infty }}\end{array}\right]}^{m}\exp \left[\begin{array}{c}\frac{-{E}_{a}}{\kappa T}\end{array}\right],\end{array}$$

14 $$w\frac{\partial N}{\partial z}+\tilde{w}\frac{\partial N}{\partial z}+N\frac{\partial \tilde{w}}{\partial z}={D}_{n}\frac{{\partial }^{2}N}{\partial {z}^{2}},$$

upon the extra informations

15 $$u=r{a}_{1},\,v=r{\Omega }_{1},\,w=0,\,T={T}_{1},\,C={C}_{1},\,N={N}_{1},\,at\,z=0,$$

16 $$u=r{a}_{2},\,v=r{\Omega }_{2},\,w=0,\,T={T}_{2},\,C={C}_{2},\,N={N}_{2}\,at\,z=H,$$

notice that the constituents of velocity are *u*(*r, ϑ, z*), *v*(*r, ϑ, z*) and *w*(*r, ϑ, z*). *σ*_*f*_ is the electrical conductivity of nanofluid, *B* = (0, 0, *B*_0_) is the magnet environment and *k*_0_ stands for the porosity of space. *Q*_0_ is the heat source/sink coefficient, *m* is the fitted rate constant such that (−1 < *m* < 1), *E*_*a*_ is the activation energy in which *a* is positive dimensional constant, *κ* = 8.61 × 10^−5^ eV/K is the Boltzmann constant and $${k}_{r}^{2}(C-{C}_{2})\,{\left[\begin{array}{c}\frac{T}{{T}_{\infty }}\end{array}\right]}^{m}\exp \frac{-{E}_{a}}{\kappa T}$$ is the modified Arrhenius term. $$\tilde{w}=\left(\begin{array}{c}\frac{b{W}_{c}}{\varDelta C}\end{array}\right)\frac{\partial C}{\partial z}$$ is the velocity component of the vector $\tilde{v}$ in *z*-side.

Introduced transformations are^23–25^

17 $$\begin{array}{c}u=r{\Omega }_{1}f{\prime} (\zeta ),\,v=r{\Omega }_{1}g(\zeta ),\,w=-2H{\Omega }_{1}f(\zeta ),\,\theta (\zeta )=\frac{T-{T}_{2}}{{T}_{1}-{T}_{2}},\,\phi (\zeta )=\frac{C-{C}_{2}}{{C}_{1}-{C}_{2}},\\ h(\zeta )=\frac{N-{N}_{2}}{{N}_{1}-{N}_{2}},\,P={\rho }_{f}{\Omega }_{1}{\nu }_{f}\left(\begin{array}{c}P(\zeta )+\frac{{r}^{2}\varepsilon }{2{H}^{2}}\end{array}\right),\,\zeta =\frac{z}{H},\end{array}$$

where ${\nu }_{f}=\frac{{\mu }_{f}}{{\rho }_{f}}$ manifests the movement viscousness and $\epsilon$ is the force per unit area representative.

Equation (17) at once justifies the preservation of quantity of matter Eq. (8). Substituting the assignments from Eq. (17) for Eqs. (9–16)

18 $$f{\prime\prime} {\prime} +\mathrm{Re}\left(\begin{array}{c}2ff{\prime\prime} -f{{\prime} }^{2}+{g}^{2}-Mf{\prime} -\frac{1}{\lambda }f{\prime} \end{array}\right)-\epsilon =0,$$

19 $$g{\prime\prime} +\mathrm{Re}\left(\begin{array}{c}2fg{\prime} -Mg{\prime} -\frac{1}{\lambda }g\end{array}\right)=0,$$

20 $$P{\prime} =\frac{2}{\lambda }f{\prime} -4\mathrm{Re}ff{\prime} -f{\prime\prime} ,$$

21 $$\theta {\prime\prime} +{\Pr }{Re}[\begin{array}{c}2f\theta {\prime} +MEc\,(\begin{array}{c}{(f{\prime} )}^{2}+{g}^{2}\end{array})\end{array}]+Nb\theta {\prime} \phi {\prime} +Nt{(\theta {\prime} )}^{2}+\gamma \theta =0,$$

22 $$\phi {\prime\prime} +{Re}\left(\begin{array}{c}2Lef\phi {\prime} +\frac{Nt}{Nb}\theta {\prime} \end{array}\right)+{\gamma }_{1}{({\gamma }_{2}\theta +1)}^{m}\phi \,\exp \,\left(\begin{array}{c}\frac{-E}{{\gamma }_{2}\theta +1}\end{array}\right)=0,$$

23 $$h{\prime\prime} +{Re}[\begin{array}{c}2Scfh{\prime} +Pe(h{\prime} \phi {\prime} -h\phi {\prime\prime} )\end{array}]=0,$$

24 $$f=0,\,f{\prime} ={k}_{1},\,g=1,\,\theta =1,\,\phi =1,\,h=1,\,P=0\,at\,\zeta =0,$$

25 $$f=0,\,f{\prime} ={k}_{2},\,g=\Omega ,\,\theta =0,\,\phi =0,\,h=0\,at\,\zeta =1,$$

notice that prime (′) represents the differentiability on behalf of *ζ*. $\Omega =\frac{{\Omega }_{2}}{{\Omega }_{1}}$ is the rotation representative, $\mathrm{Re}=\frac{{\Omega }_{1}{H}^{2}}{{\nu }_{f}}$ manifests the Reynolds quantity, $M=\frac{{\sigma }_{f}{B}_{0}^{2}}{{\rho }_{f}{\Omega }_{1}}$ represents the magnetic field parameter, $\lambda =\frac{{k}_{0}{\Omega }_{1}}{{\nu }_{f}}$ manifests the porosity representative, $\Pr =\frac{{(\rho {c}_{P})}_{f}{\nu }_{f}}{\alpha }$ denotes the Prandtl quantity and $Ec=\frac{{r}^{2}{\Omega }_{1}^{2}}{{c}_{P}({T}_{1}-{T}_{2})}$ is the Eckert quantity, $Le=\frac{{\nu }_{f}}{{D}_{B}}$ represents the Levis representative, $Sc=\frac{{\nu }_{f}}{{D}_{n}}$ represents the Schmidt representative, and $Pe=\frac{b{W}_{c}}{{D}_{n}}$ represents the Peclet representative. The scaled stretching parameters are defined as ${k}_{1}=\frac{{a}_{1}}{{\Omega }_{1}}$, and ${k}_{2}=\frac{{a}_{2}}{{\Omega }_{1}}$. $Nb=\frac{{D}_{B}({C}_{2}-{C}_{1})}{{\nu }_{f}}$ manifests the random movement representative, $Nt=\frac{\tau {D}_{T}({T}_{2}-{T}_{1})}{{\nu }_{f}{T}_{1}}$ represents the thermophoresis representative. $\gamma =\frac{{Q}_{0}}{{\Omega }_{1}{(\rho {c}_{P})}_{f}}$, ${\gamma }_{1}=\frac{{k}_{r}^{2}{H}^{2}}{{\nu }_{f}}$, ${\gamma }_{2}=\frac{{T}_{1}-{T}_{2}}{{T}_{2}}$ and $E=\frac{{E}_{a}}{\kappa {T}_{2}}$ are the heat source/sink, chemical reaction, temperature difference and non-dimensional activation energy parameters respectively.

Upon differentiability of Eq. (18) on behalf of *ζ*, the equation accomplishes as

26 $$f{\prime\prime} {\prime\prime} +\mathrm{Re}\left(\begin{array}{c}2ff{\prime\prime} {\prime} +2gg{\prime} -Mf{\prime\prime} -\frac{1}{\lambda }f{\prime\prime} \end{array}\right)=0,$$

Attaining the solution for Eq. (18) and Eqs. (24–25), the force per unit area representative $\epsilon$ is evaluated like

27 $$\epsilon =f{\prime\prime} {\prime} (0)-\mathrm{Re}\left[\begin{array}{c}{(f{\prime} (0))}^{2}-{(g(0))}^{2}+Mf{\prime} (0)+\frac{1}{\lambda }f{\prime} (0)\end{array}\right],$$

Applying inverse process of differentiation on Eq. (20) on behalf of *ζ* and including the limits as zero to *ζ* on account of achieving the quantity *P* as

28 $$P=-2\left[\begin{array}{c}\mathrm{Re}\left(\begin{array}{c}{(f)}^{2}+\frac{1}{\lambda }{\int }_{0}^{\zeta }\,f\end{array}\right)+(f{\prime} -f{\prime} (0))\end{array}\right],$$

## Computation methodology

Applying optimal homotopy analysis method (OHAM)^22,37^, the starting approximations and helping linear quantities exists as

29 $$\begin{array}{c}{f}_{0}(\zeta )={k}_{1}\zeta -(2{k}_{1}+{k}_{2}){\zeta }^{2}+({k}_{1}+{k}_{2}){\zeta }^{3},\,{g}_{0}(\zeta )=1-\zeta +\Omega \zeta ,\\ \,{\theta }_{0}(\zeta )\,=1-\zeta ,\,{\phi }_{0}(\zeta )=1-\zeta ,\,{h}_{0}(\zeta )=1-\zeta ,\end{array}$$

30 $${{\boldsymbol{L}}}_{f}=f{\prime\prime} {\prime\prime} ,\,{{\boldsymbol{L}}}_{g}=g{\prime\prime} ,\,{{\boldsymbol{L}}}_{\theta }=\theta {\prime\prime} ,\,{{\boldsymbol{L}}}_{\varphi }=\phi {\prime\prime} ,\,{{\boldsymbol{L}}}_{h}=h{\prime\prime} $$

characterizing

31 $$\begin{array}{c}{{\boldsymbol{L}}}_{f}[\begin{array}{c}{C}_{1}+{C}_{2}\zeta +{C}_{3}{\zeta }^{2}+{C}_{4}{\zeta }^{3}\end{array}]=0,\,{{\boldsymbol{L}}}_{g}[\begin{array}{c}{C}_{5}+{C}_{6}\zeta \end{array}]=0,\,{{\boldsymbol{L}}}_{\theta }[\begin{array}{c}{C}_{7}+{C}_{8}\zeta \end{array}]=0,\\ {{\boldsymbol{L}}}_{\phi }[\begin{array}{c}{C}_{9}+{C}_{10}\zeta \end{array}]=0,\,{{\boldsymbol{L}}}_{h}[\begin{array}{c}{C}_{11}+{C}_{12}\zeta \end{array}]=0,\end{array}$$

evidently *C*_*i*_(*i* = 1–12) are known as the randomly chosen quantities.

## Outcomes

Outputs are assembled for the simplified statements in Eqs. (19, 21–26) accompanying the assisting informations in Eqs. (24–25) under the usage of MATHEMATICA. The potentialities of linked representatives on the respective profiles are displayed in Figs. (2–38) and Figs. (39–47). Physical sketch of the problem is presented in Fig. 1.

**Figure 2:**
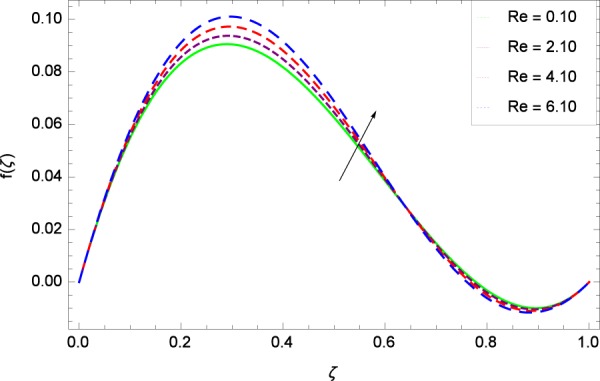
Axisymmetric movement graph with exceeding values of *Re*.

**Figure 3:**
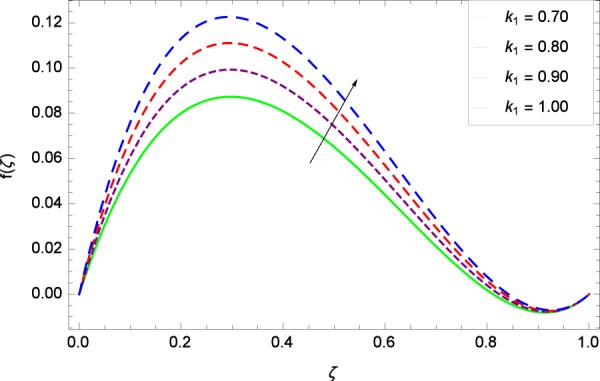
Axisymmetric movement graph with exceeding values of *k*_1_.

**Figure 4:**
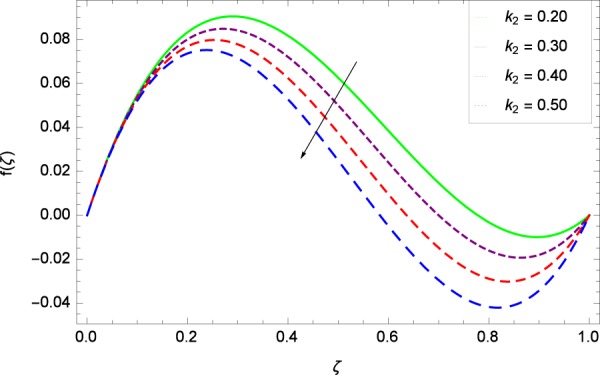
Axisymmetric movement graph with exceeding values of *k*_2_.

**Figure 5:**
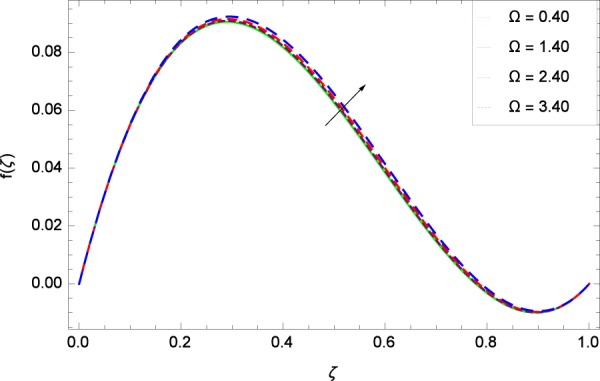
Axisymmetric movement graph with exceeding values of Ω.

**Figure 6:**
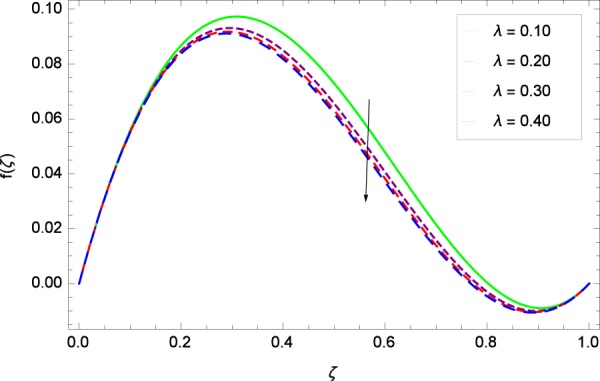
Axisymmetric movement graph with exceeding values of *λ*.

**Figure 7:**
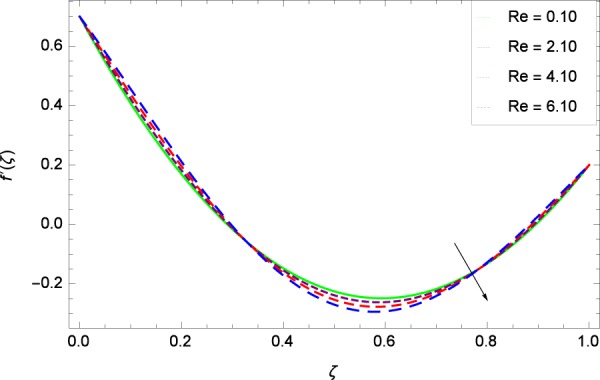
Axisymmetric movement graph with exceeding values of *Re*.

**Figure 8:**
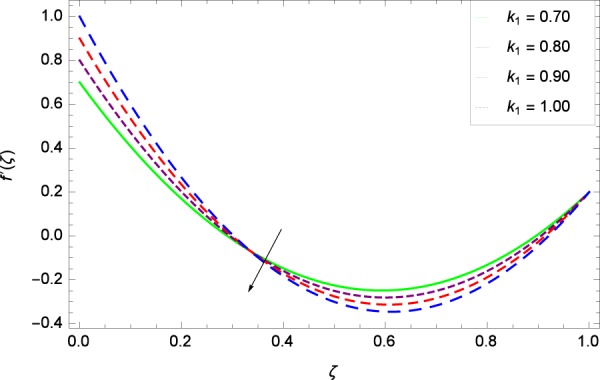
Axisymmetric movement graph with exceeding values of *k*_1_.

**Figure 9:**
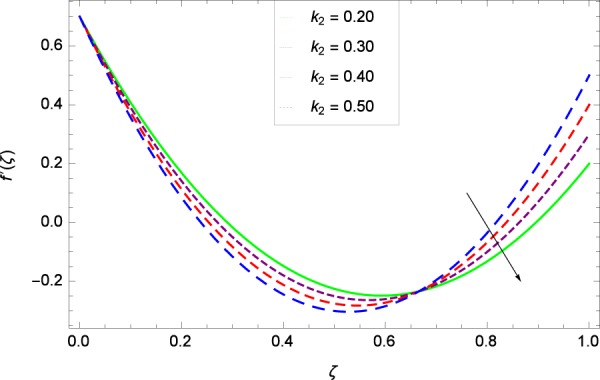
Axisymmetric movement graph with exceeding values of *k*_2_.

**Figure 10:**
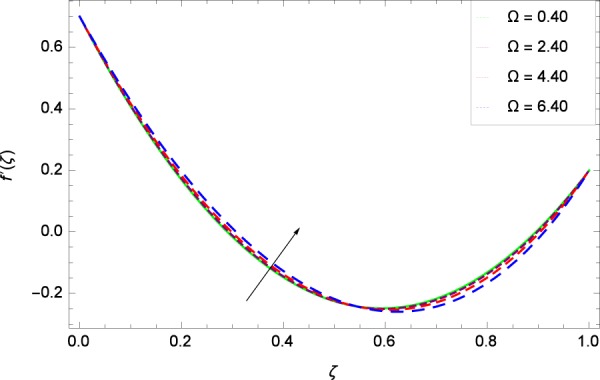
Axisymmetric movement graph with exceeding values of Ω.

**Figure 11:**
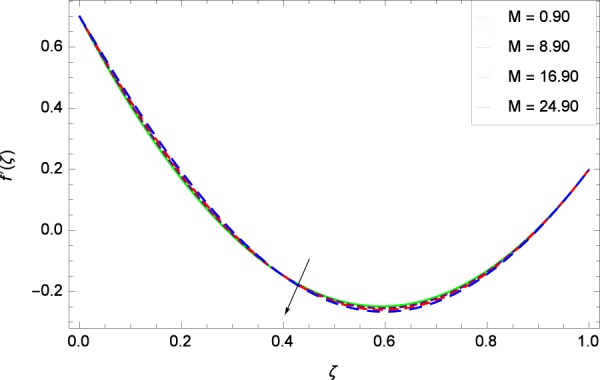
Axisymmetric movement graph with exceeding values of *M*.

**Figure 12:**
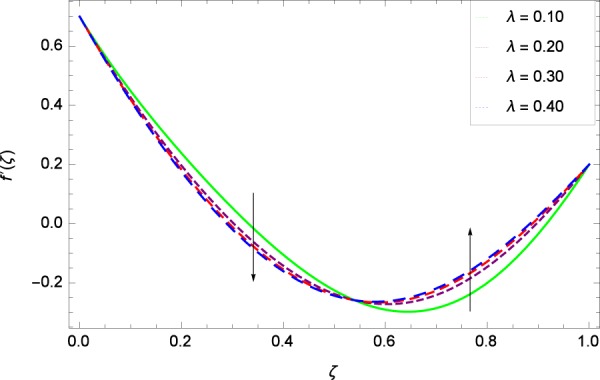
Axisymmetric movement graph with exceeding values of *λ*.

**Figure 13:**
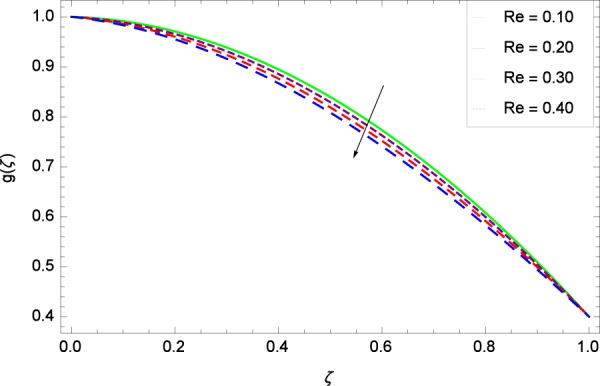
Axisymmetric movement graph with exceeding values of *Re*.

**Figure 14:**
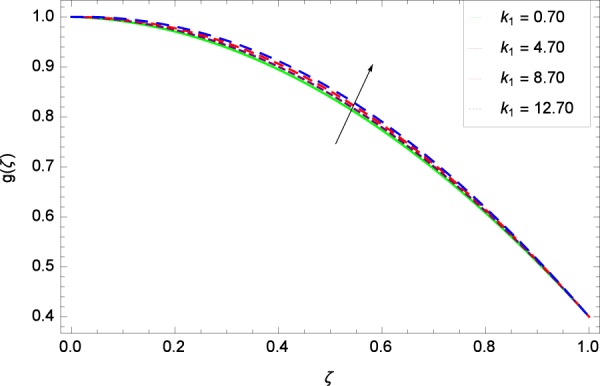
Axisymmetric movement graph with exceeding values of *k*_1_.

**Figure 15:**
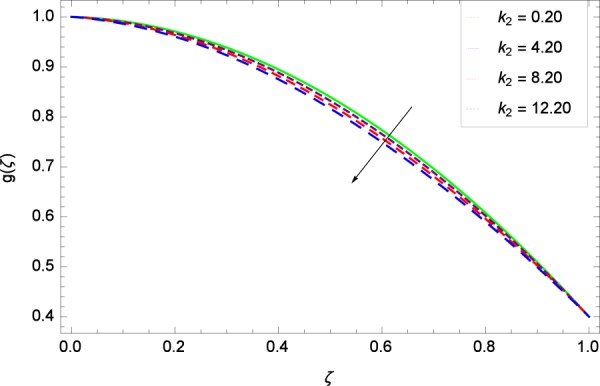
Axisymmetric movement graph with exceeding values of *k*_2_.

**Figure 16:**
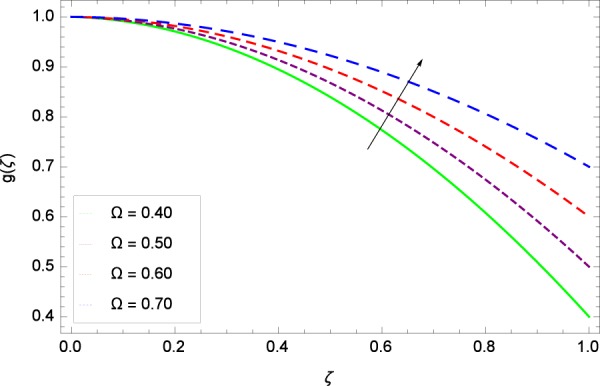
Axisymmetric movement graph with exceeding values of Ω.

**Figure 17:**
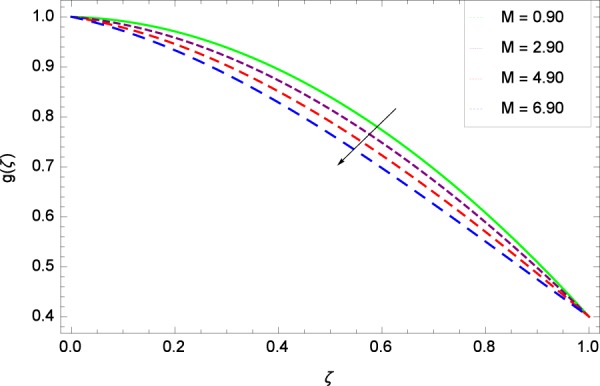
Axisymmetric movement graph with exceeding values of *M*.

**Figure 18:**
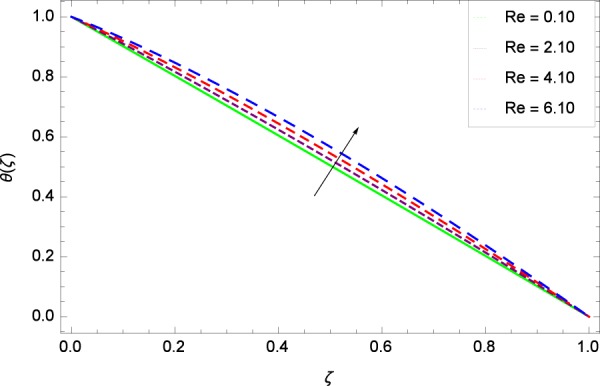
Temperature graph with exceeding values of *Re*.

**Figure 19:**
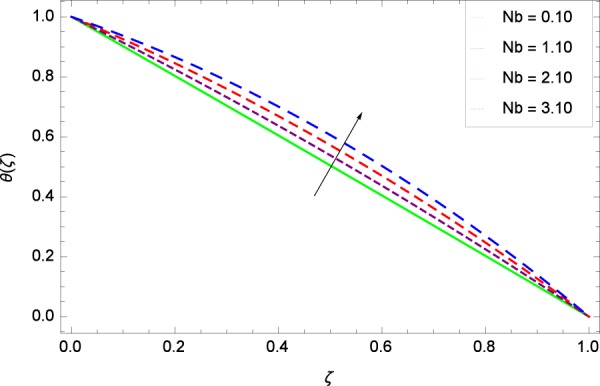
Temperature graph with exceeding values of *Nb*.

**Figure 20:**
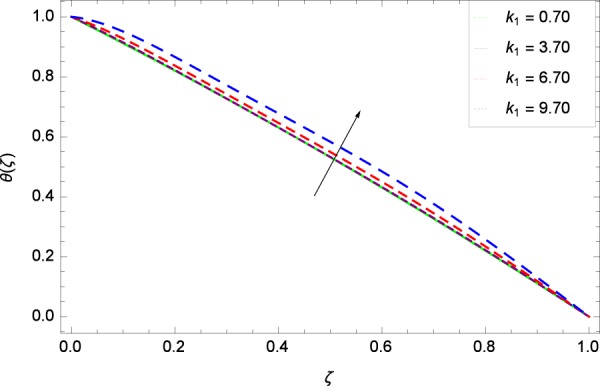
Temperature graph with exceeding values of *k*_1_.

**Figure 21:**
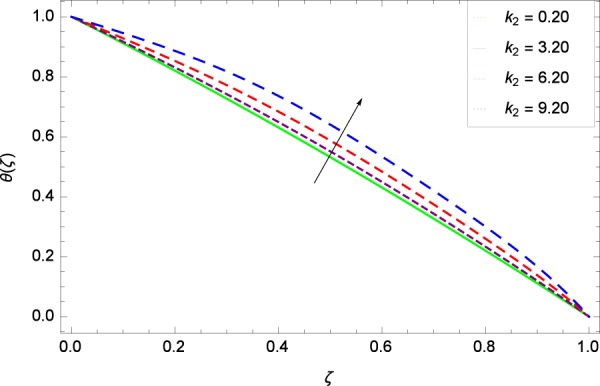
Temperature graph with exceeding values of *k*_2_.

**Figure 22:**
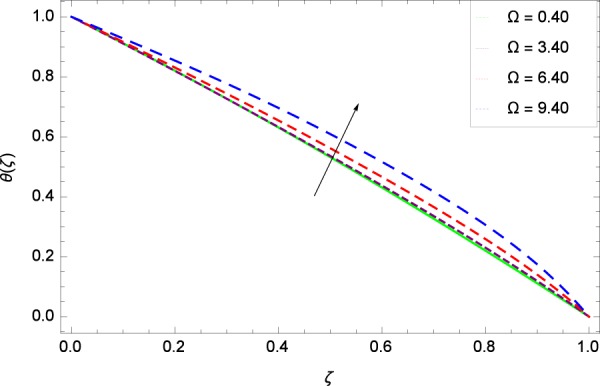
Temperature graph with exceeding values of Ω.

**Figure 23:**
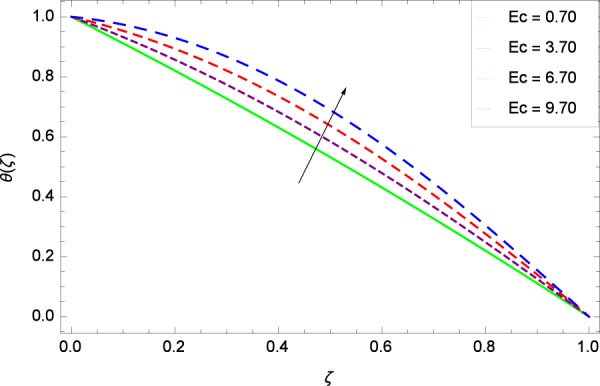
Temperature graph with exceeding values of *Ec*.

**Figure 24:**
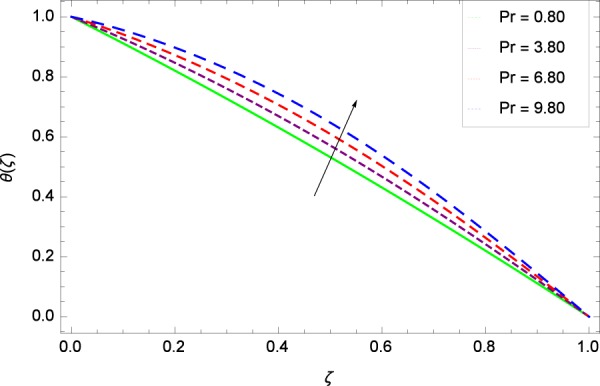
Temperature graph with exceeding values of *Pr*.

**Figure 25:**
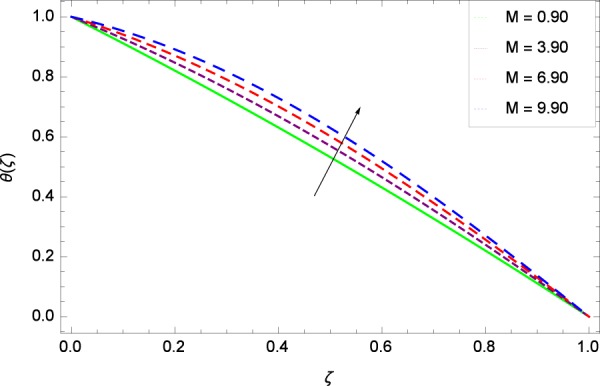
Temperature graph with exceeding values of *M*.

**Figure 26:**
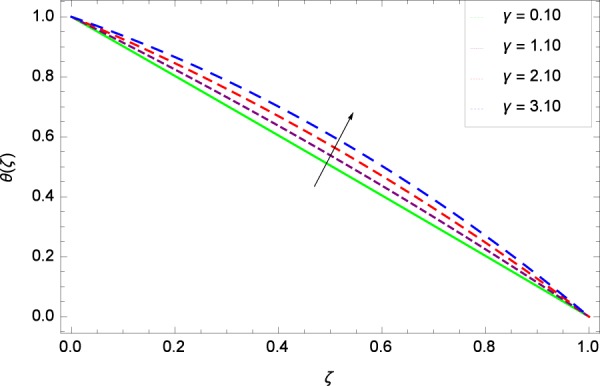
Temperature graph with exceeding values of *γ*.

**Figure 27:**
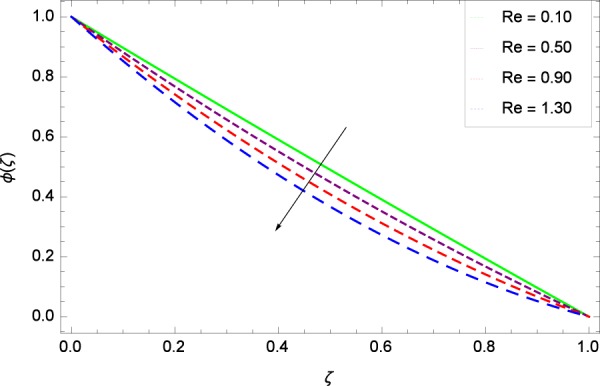
Concentration graph with exceeding values of *Re*.

**Figure 28:**
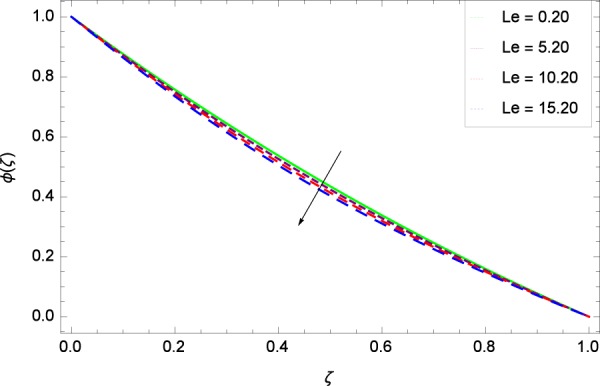
Concentration graph with exceeding values of *Le*.

**Figure 29:**
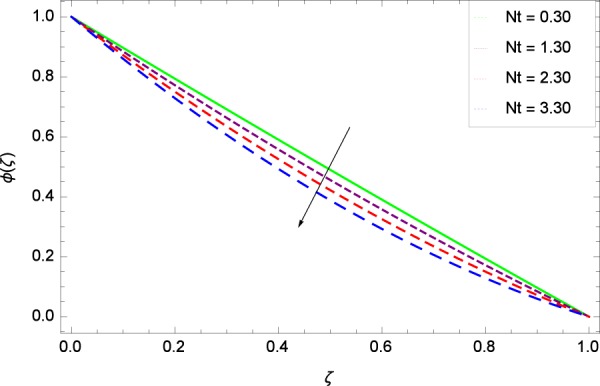
Concentration graph with exceeding values of *Nt*.

**Figure 30:**
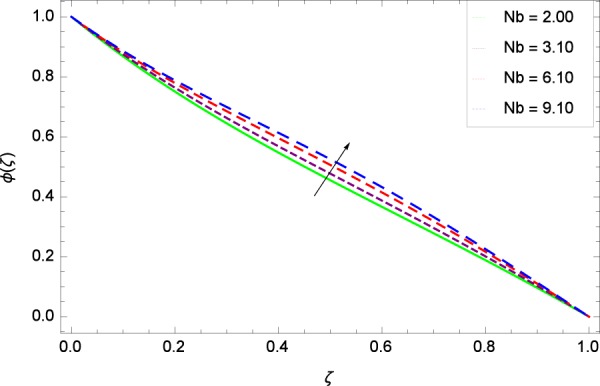
Concentration graph with exceeding values of *Nb*.

**Figure 31:**
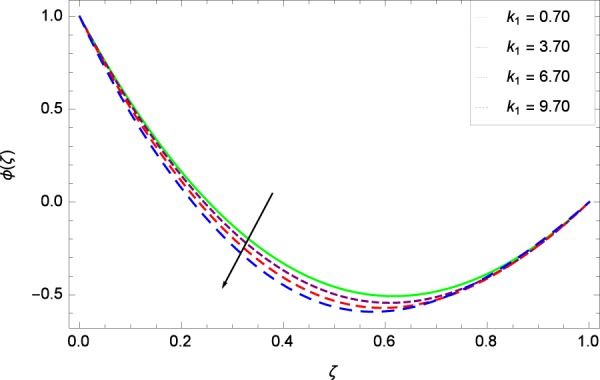
Concentration graph with exceeding values of *k*_1_.

**Figure 32:**
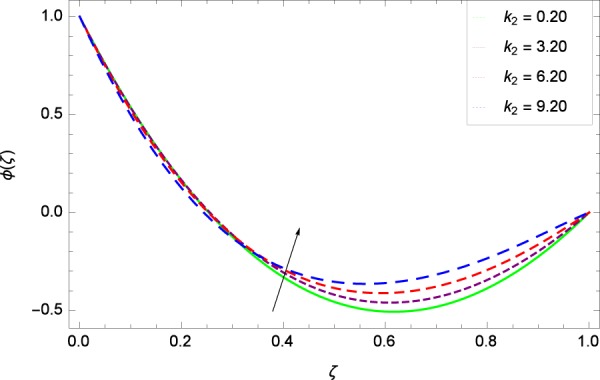
Concentration graph with exceeding values of *k*_2_.

**Figure 33:**
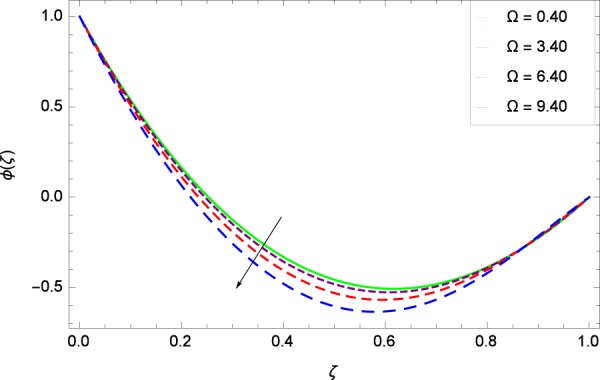
Concentration graph with exceeding values of Ω.

**Figure 34:**
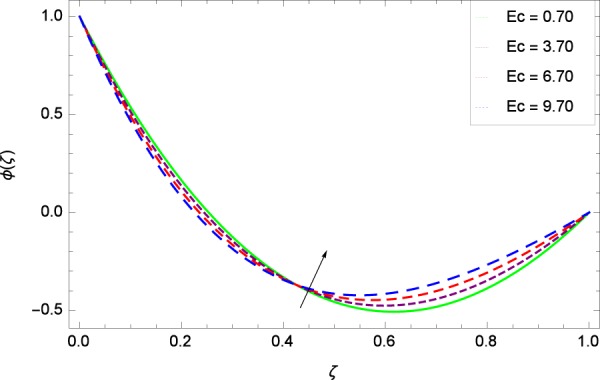
Concentration graph with exceeding values of *Ec*.

**Figure 35:**
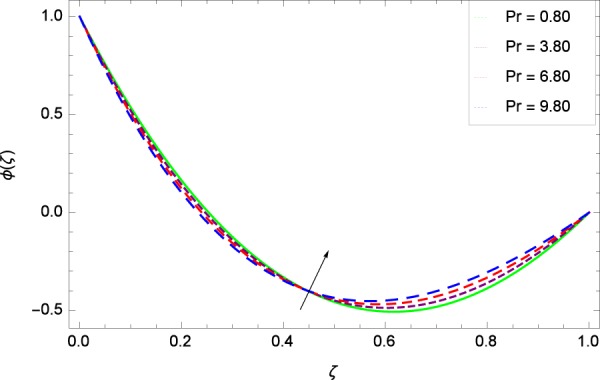
Concentration graph with exceeding values of *Pr*.

**Figure 36:**
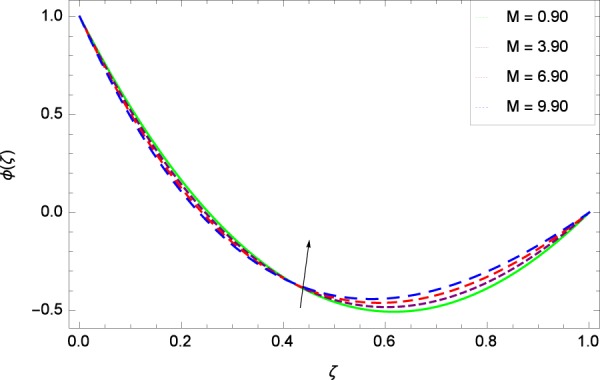
Concentration graph with exceeding values of *M*.

**Figure 37:**
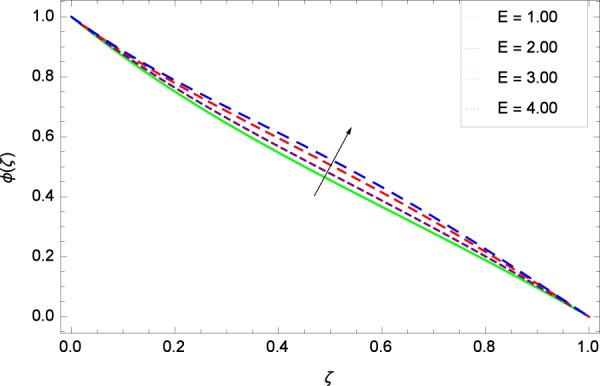
Concentration graph with exceeding values of *E*.

**Figure 38:**
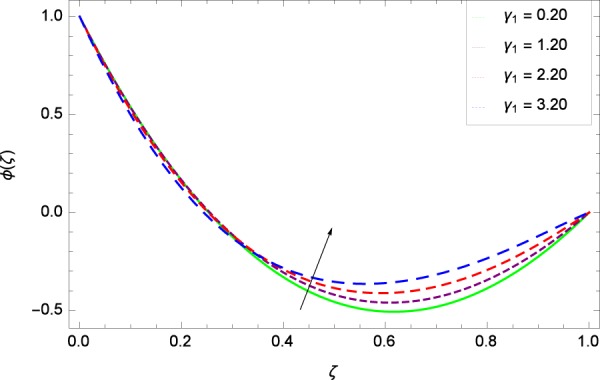
Concentration graph with exceeding values of *γ*_1_.

**Figure 39:**
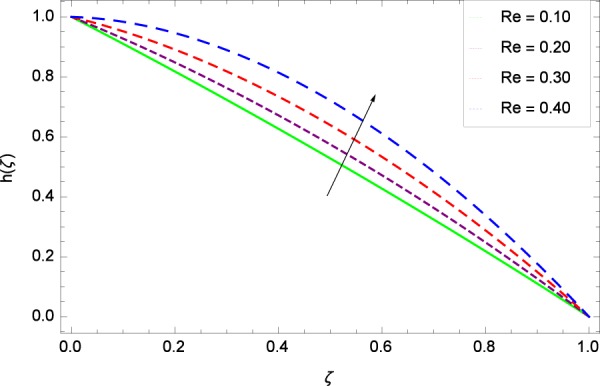
Motile microorganisms concentration graph with exceeding values of *Re*.

**Figure 40:**
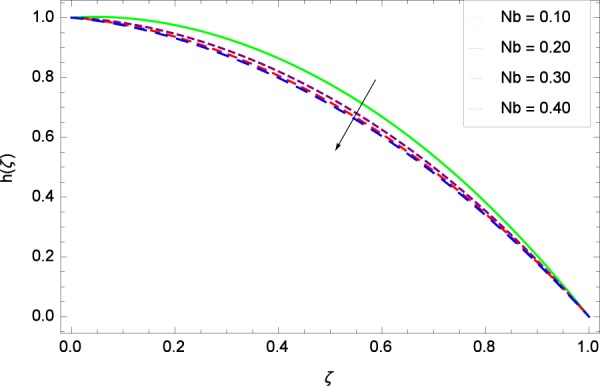
Motile microorganisms concentration graph with exceeding values of *Nb*.

**Figure 41:**
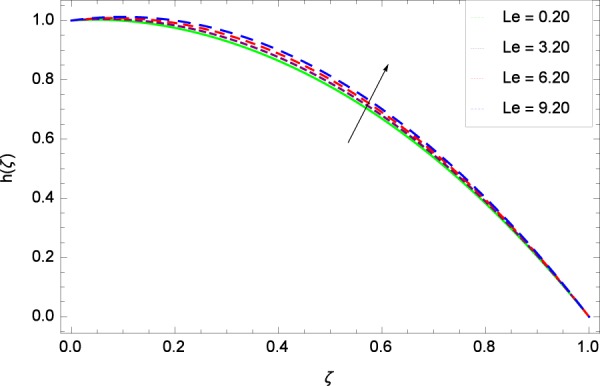
Motile microorganisms concentration graph with exceeding values of *Le*.

**Figure 42:**
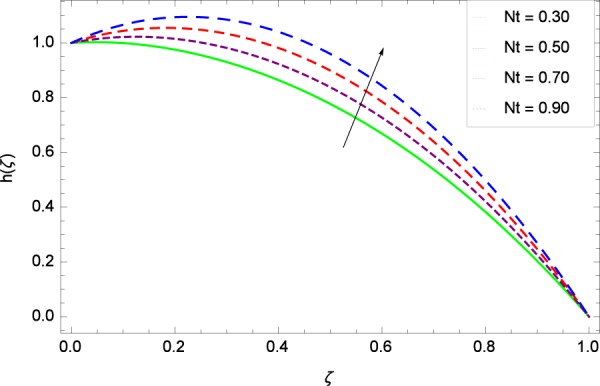
Motile microorganisms concentration graph with exceeding values of *Nt*.

**Figure 43:**
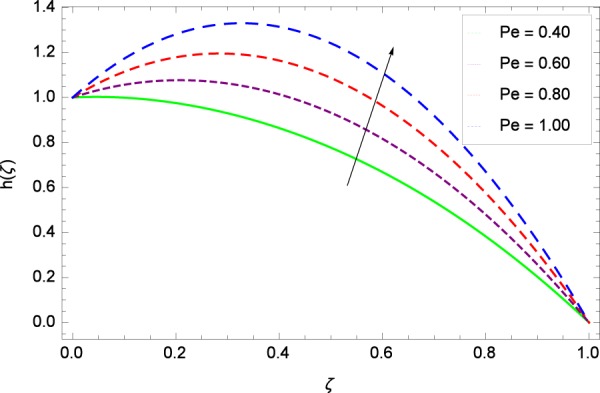
Motile microorganisms concentration graph with exceeding values of *Pe*.

**Figure 44:**
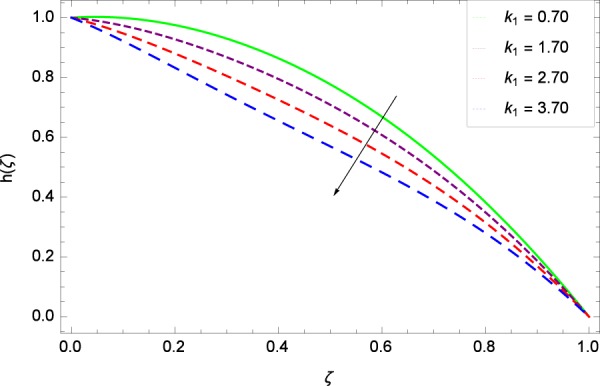
Motile microorganisms concentration graph with exceeding values of *k*_1_.

**Figure 45:**
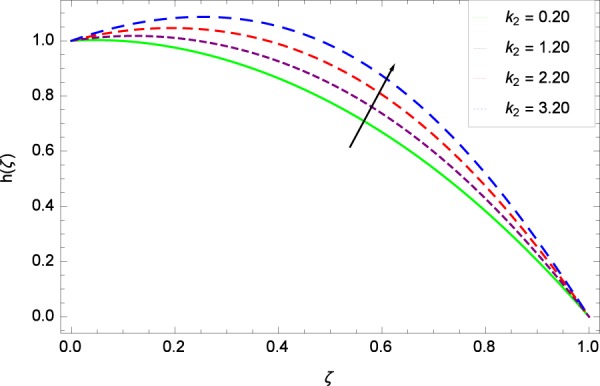
Motile microorganisms concentration graph with exceeding values of *k*_2_.

**Figure 46:**
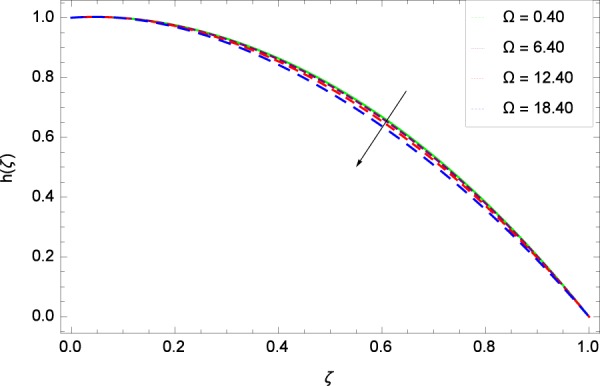
Motile microorganisms concentration graph with exceeding values of Ω.

**Figure 47:**
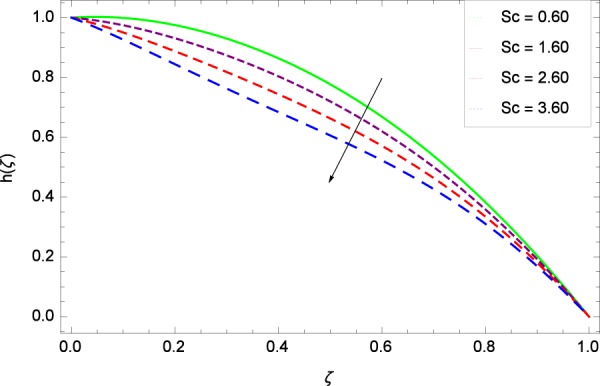
Motile microorganisms concentration graph with exceeding values of *Sc*.

### Axial velocity profile

Figure 2 shows that velocity distribution *f*(*ζ*) has a diminishing behavior for larger values of Reynolds number *Re*. Higher quantities of *Re* indicate the reduced flow rate. Figure 3 shows that the axial movement *f*(*ζ*) increases due to *k*_1_ while the opposite trend for velocity *f*(*ζ*) is observed in Fig. 4 for increasing the *k*_2_ since in this way stretching for the flow is decreased, consequently, the boundary layer thickness is made low. Figure 5 exhibits all the assigned values of Ω and axial velocity *f*(*ζ*) which shows the successful completion of their effects. Physically, the velocity is partially shifted on account of swirling. Figure 6 shows that on establishing porous medium to the fluid flow, the velocity *f*(*ζ*) is decreased. The fact is that the presence of porous medium with gradually increasing values increase the resistance in flow of fluid which boosts friction close to the wall, therefore, the velocity is diminished and the boundary layer is made thin. For *λ* = 0, the system becomes when the fluid does not saturate the porous space.

### Radial velocity profile

Figure 7 displays that the velocity component *f*’(*ζ*) decreases owing to strong impacts of Reynolds number *Re*. Figure 8 demonstrates that *f*’(*ζ*) reliant to radial direction declines for numerous values of stretching parameter *k*_1_. Physically, an enhancement in *k*_1_ depicts that the radial component of velocity field is less dominant in the present rotating flow. The effect of stretching parameter *k*_2_ on *f*’(*ζ*) is shown in Fig. 9. It provides that velocity distribution is smaller with an increment in *k*_2_. It is felt that radially motion *f*’(*ζ*) accelerates with rotation quantity Ω in Fig. 10 which offers the significance recognition of the present work. Figure 11 shows that magnetic field parameter *M* is associated with low level of velocity. Lorentz forces are produced due to the existence of magnetic field which ultimately resist the flow. When *M* = 0, the study becomes of hydrodynamic nature. Figure 12 is related to the porous medium parameter *λ* and the radial velocity *f*’(*ζ*). The flow is concerned to the dual nature. For 0.0 ≤ *ζ* ≤ 0.5, the velocity *f*’(*ζ*) is decreased but when the *ζ* crosses the value of 0.50, the flow is of increasing behavior.

### Tangential velocity profile

Figure 13 is showing the effect of Reynolds number *Re* on tangential velocity *g*(*ζ*). It is perceived that for improving values of *Re*, the graph shows a decreasing behavior. In Fig. 14, tangential velocity *g*(*ζ*) is decreased with increasing the stretching parameter *k*_1_. Figure 15 witnesses that the tangential velocity *g*(*ζ*) shifts to the effective decreasing results with the stretching parameter *k*_2_. A decay of the momentum boundary layer is observed. Figure 16 points out that the rotation parameter Ω increases the tangential velocity *g*(*ζ*). Figure 17 projects that for the digital values 0.90, 2.90, 4.90, and 6.90 of *M*, the magnetic field is taking over the control to reduce the tangential velocity.

### Temperature profile

Figure 18 shows the maximization of temperature *θ*(*ζ*) and Reynolds number *Re*. This improvement in heat transfer is physically attributed as increasing values of *Re* result in enhancement of thickness of the fluid which surges the temperature. Brownian motion parameter *Nb* and temperature *θ*(*ζ*) in Fig. 19 show that upon increasing *Nb*, the improvement is made in heat transfer. In Brownian motion, the particles kinetic energy increases due to the collision hence temperature is made high. In Fig. 20, the temperature *θ*(*ζ*) shows high values due to its ability to get the values of the stretching parameter *k*_1_. Figure 21 is shown for the respective choices of stretching parameter *k*_2_ and for temperature *θ*(*ζ*). It is just needed to fill the gape through values of *k*_2_ and increase the temperature. The rotation parameter Ω generates extra heating to the system in Fig. 22. Temperature *θ*(*ζ*) is increased just on increasing the parameter Ω. The greater values of *Ec* are used to access the enhanced temperature *θ*(*ζ*) in Fig. 23. The agent *Ec* assigns the values to a concerned system. It is seen that temperature increases against the quantities of *Ec*. It is a fact that Eckert number is a ratio of enthalpy difference and kinetic energy. That’s why temperature increases for the greater values of *Ec*. The system gets the parameter *Pr* feeding the designated values 0.80, 3.80, 6.80 and 9.80 during swirling to enhance the temperature shown through Fig. 24. The temperature *θ*(*ζ*) is changed to highest level after the high status of magnetic field parameter *M* as shown in Fig. 25. Due to the application of magnetic field, the Lorentz forces result in the good movement of molecular movement of nanoparticles, increasing *θ*(*ζ*). Figure 26 shows the effect of heat generation/absorption parameter *γ* on temperature *θ*(*ζ*) which shows that temperature increases with increasing values of *γ*. Note that the *γ* values greater than zero represents the heat generation and *γ* values less than zero shows the heat absorption parameter.

### Nanoparticles concentration

It is observed that nanoparticles concentration *ϕ*(*ζ*) is decreasing with the increasing values of Reynolds number *Re* in Fig. 27. *ϕ*(*ζ*) is decreased when the Lewis number *Le* is enhanced for the positive values as demonstrated in Fig. 28. The reason is that the given values decrease the diffusion of concentration. Figure 29 shows that the thermophoresis parameter *Nt* decreases the nanoparticles concentration *ϕ*(*ζ*). In Fig. 30, Brownian motion parameter *Nb* enhances the nanoparticle concentration *ϕ*(*ζ*). Physically, higher values of *Nb* retain the small amount of viscous force and larger coefficient of Brownian diffusion so the temperature enhances which improves the concentration. The stretching parameter *k*_1_ enhances the concentration *ϕ*(*ζ*) by data 0.70, 3.70, 6.70, and 9.70 as demonstrated in Fig. 31. Another stretching parameter *k*_2_ provides the results in Fig. 32 in which the concentration *ϕ*(*ζ*) is changed to the high level. The rotation parameter Ω is used to see the changes made in the concentration *ϕ*(*ζ*) through Fig. 33. Concentration is made weak through rotation. Eckert number *Ec* provides the enhanced saturation of nanoparticles as shown through Fig. 34. Figure 35 shows that concentration *ϕ*(*ζ*) is promoted to high stage due to the parameter *Pr*. The magnetic field parameter *M* also helps to strengthen the enhancement of nanoparticles saturation shown through Fig. 36. Figure 37 depicts that nanoparticles concentration enhances with the non-dimensional activation energy parameter *E*. Equation (22) shows the strong coupling of the nanoparticle concentration *ϕ* with ${\gamma }_{1}{({\gamma }_{2}\theta +\mathrm{1)}}^{m}$ and $$exp\left(\begin{array}{c}\frac{-E}{{\gamma }_{2}\theta +1}\end{array}\right)$$. So if the activation energy rises, the nanoparticles concentration is easily enhanced. Physically, it is due to the fact that due to activation energy, the system gets an extra energy which enhances the chemical reaction and hence the concentration. Figure 38 reveals that the nanoparticles concentration is enhanced with the greater values of chemical reaction parameter *γ*_1_.

### Motile microorganisms concentration

Figure 39 depicts that gyrotactic microorganisms flow is high under the excessive values of *Re*. Figure 40 is about the parameter *Nb* and motile microorganisms concentration *h*(*ζ*). Physically, Brownian motion has effect on the random movement of the nanoparticles. So in the presence of gyrotactic microorganisms, the parameter *Nb* has the leading role in developing *h*(*ζ*). In Fig. 41, the Lewis number *Le* corresponds to the higher motile microorganisms concentration *h*(*ζ*). Figure 42 represents that motile microorganisms concentration *h*(*ζ*) for the larger values of thermophoresis parameter *Nt*. An enhancement in *Nt* provides the substantial thermophoretic force on account of which nanoparticles transfer to lower energy state level thereby microorganisms concentration becomes high. Motile microorganisms concentration *h*(*ζ*) reach to the peak point for the prescribed values of Peclet number *Pe* in Fig. 43. The inspection of the performance of *Pe* with respect to (*h*(*ζ*)) is easily confirmed from Eq. (23). It is witnessed that as *Pe* is attempting to resume positive values, event causes *h*(*ζ*) to high position. In Fig. 44, as the stretching parameter *k*_1_ begins to 0.70 until 3.70, motile microorganisms concentration *h*(*ζ*) drops down while in Fig. 45, motile microorganisms concentration *h*(*ζ*) is associated to the high values of stretching parameter *k*_2_ which has positively influenced the *h*(*ζ*). The rotation parameter Ω shows a weaker diffusivity of microorganisms in Fig. 46. Figure 47 visualized the decreasing phenomena of motile microorganisms concentration *h*(*ζ*) due to the variation in Schmidt number *Sc*. Probably, the abundance of *Sc*, the concentration *h*(*ζ*) stops to nurturing.

## Conclusions

Analytical analysis is addressed to the Buongiorno’s nanofluid model for stretchable rotating disks with gyrotactic microorganisms flow, porous medium, Brownian motion and thermophoresis, heat source/sink, Arrhenius activation energy and binary chemical reaction. Optimal homotopy analysis method (OHAM) is applied for the solution which is shown through graphs for the interesting effects of all the embedded parameters. Possible future work is to investigate the non-Newtonian and hybrid nanofluids for rotating systems under different boundary conditions.

## Data Availability

All the relevant material is available.
