# Author Correction: A comprehensive study to the assessment of Arrhenius activation energy and binary chemical reaction in swirling flow

**DOI:** 10.1038/s41598-020-76587-0

**Published:** 2020-11-09

**Authors:** Noor Saeed Khan, Zahir Shah, Meshal Shutaywi, Poom Kumam, Phatiphat Thounthong

**Affiliations:** 1 Department of Mathematics, Abdul Wali Khan University, Mardan, 23200 Khyber Pakhtunkhwa Pakistan; 2 Department of Mathematics, College of Science & Arts, King Abdulaziz University, P. O. Box 344, Rabigh, 21911 Saudi Arabia; 3 KMUTT Fixed Point Research Laboratory, Room SCL 802 Fixed Point Laboratory, Science Laboratory Building, Department of Mathematics, Faculty of Science, King Mongkut’s University of Technology Thonburi (KMUTT), Bangkok, 10140 Thailand; 4 KMUTT-Fixed Point Theory and Applications Research Group, Theoretical and Computational Science Center (TaCS), Science Laboratory Building, Faculty of Science, King Mongkut’s University of Technology Thonburi (KMUTT), Bangkok, 10140 Thailand; 5 Department of Medical Research, China Medical University Hospital, China Medical University, Taichung, 40402 Taiwan; 6 Renewable Energy Research Centre, Department of Teacher Training in Electrical Engineering, Faculty of Technical Education, King Mongkut’s University of Technology North Bangkok, 1518, Wongsawang, Bangsue, 10800 Bangkok, Thailand

Correction to: *Scientific Reports* https://doi.org/10.1038/s41598-020-64712-y, published online 12 May 2020

In the original version of this Article, Meshal Shutaywi was incorrectly affiliated with ‘Department of Mathematics College of Science & Arts, Rabigh King Abdul-Aziz University, Rabigh, 21911, Saudi Arabia’. The correct affiliation is listed below.

Department of Mathematics, College of Science & Arts, King Abdulaziz University, P. O. Box 344, Rabigh, 21911, Saudi Arabia

Additionally, the original version of this Article contained several typographical errors.

In the Methods section, under the subheading ‘Computational methodology’ in Equation 30,

$$\user2{L}_{f} ~ = f{^{\prime\prime\prime\prime}},\;\user2{L}_{g} = g{^{\prime\prime}}, \user2{L}_{\theta } = \theta {^{\prime\prime}} , \user2{L}_{\varphi } ~ = \phi{^{\prime\prime}},\user2{L}_{h} ~ =h{^{\prime\prime}}$$

now reads:

$$\user2{L}_{f} ~ = f{^{\prime\prime\prime\prime}},\;\user2{L}_{g} = g{^{\prime\prime}}, \user2{L}_{\theta } = \theta {^{\prime\prime}} , \user2{L}_{\phi } ~ = \phi{^{\prime\prime}},\user2{L}_{h} ~ =h{^{\prime\prime}}$$

In the Outcomes section, under the subheading ‘Axial velocity profile’,

“Figure 2 shows that velocity distribution f(ζ) has a diminishing behavior for larger values of Reynolds number Re. Higher quantities of Re indicate the reduced flow rate.”

now reads:

“Figure 2 shows that velocity distribution f(ζ) has an increasing behavior for larger values of Reynolds number Re. Higher quantities of Re indicate the increment in flow rate.”

In the Outcome section, under the subheading ‘Nanoparticles concentration’,

“The stretching parameter *k*_1_ enhances the concentration *ϕ*(*ζ*) by data 0.70, 3.70, 6.70, and 9.70 as demonstrated in Figure 31.”

now reads:

“The stretching parameter *k*_1_ reduces the concentration *ϕ*(*ζ*) by data 0.70, 3.70, 6.70, and 9.70 as demonstrated in Figure 31.”

In the Outcome section, under the subheading ‘Motile microorganisms concentration’,

“So in the presence of gyrotactic microorganisms, the parameter *Nb* has the leading role in developing *h*(*ζ*).”

now reads:

“So in the presence of gyrotactic microorganisms, the parameter *Nb* has the leading role in decreasing *h*(*ζ*).”

Lastly, the original version of this Article contained an error in Figure 42 where the x-axis was omitted.

These errors have now been corrected in the PDF and HTML versions of the Article.

